# Application of a Human Blood Brain Barrier Organ-on-a-Chip Model to Evaluate Small Molecule Effectiveness against Venezuelan Equine Encephalitis Virus

**DOI:** 10.3390/v14122799

**Published:** 2022-12-15

**Authors:** Niloufar A. Boghdeh, Kenneth H. Risner, Michael D. Barrera, Clayton M. Britt, David K. Schaffer, Farhang Alem, Jacquelyn A. Brown, John P. Wikswo, Aarthi Narayanan

**Affiliations:** 1 Biomedical Research Laboratory, Institute of Biohealth Innovation, George Mason University, Manassas, VA 20110, USA; 2 College of Science, School of Systems Biology, George Mason University, Manassas, VA 20110, USA; 3 College of Science, Department of Biology, George Mason University, Fairfax, VA 22030, USA; 4 Vanderbilt Institute for Integrative Biosystems Research and Education, Vanderbilt University, Nashville, TN 37212, USA; 5 Department of Physics and Astronomy, Vanderbilt University, Nashville, TN 37235, USA; 6 Department of Biomedical Engineering, Vanderbilt University, Nashville, TN 37235, USA; 7 Department of Molecular Physiology and Biophysics, Vanderbilt University, Nashville, TN 37232, USA

**Keywords:** Venezuelan Equine Encephalitis Virus, alphaviruses, neurovascular unit (NVU), organ-on-a-chip, blood brain barrier, omaveloxolone, RTA408

## Abstract

The blood brain barrier (BBB) is a multicellular microenvironment that plays an important role in regulating bidirectional transport to and from the central nervous system (CNS). Infections by many acutely infectious viruses such as alphaviruses and flaviviruses are known to impact the integrity of the endothelial lining of the BBB. Infection by Venezuelan Equine Encephalitis Virus (VEEV) through the aerosol route causes significant damage to the integrity of the BBB, which contributes to long-term neurological sequelae. An effective therapeutic intervention strategy should ideally not only control viral load in the host, but also prevent and/or reverse deleterious events at the BBB. Two dimensional monocultures, including trans-well models that use endothelial cells, do not recapitulate the intricate multicellular environment of the BBB. Complex in vitro organ-on-a-chip models (OOC) provide a great opportunity to introduce human-like experimental models to understand the mechanistic underpinnings of the disease state and evaluate the effectiveness of therapeutic candidates in a highly relevant manner. Here we demonstrate the utility of a neurovascular unit (NVU) in analyzing the dynamics of infection and proinflammatory response following VEEV infection and therapeutic effectiveness of omaveloxolone to preserve BBB integrity and decrease viral and inflammatory load.

## 1. Introduction

Acutely infectious RNA viruses pose an important concern to the global community due to their potential to cause epidemics and pandemics, and the lack of effective intervention strategies against them, particularly post-exposure therapeutics. There is a significant thrust into growing the preclinical pipeline for effective intervention strategies that will support the establishment of a robust therapeutic portfolio as an integral aspect of future pandemic preparedness.

Many vector-borne infectious agents, including mosquito-transmitted viruses, such as alphaviruses, lack FDA (United States Food and Drug Administration)-approved therapeutics for treatment in spite of periodic occurrences of natural outbreaks in many parts of the world. Venezuelan Equine Encephalitis Virus (VEEV) is a new world alphavirus that is transmitted by mosquitoes, resulting in disease in equines and humans [1,2,3,4]. While the mortality rate associated with natural transmission of VEEV is low (<1%), neurological manifestations have been recorded in about 4% to 14% of the cases [1]. Fatal encephalitic outcomes are more likely in children, with a higher potential for long-term neurological sequelae. Infection of pregnant women can lead to still birth, abortions and birth defects. VEEV is also highly stable as an aerosol and retains infectivity, with aerosol transmission leading to a higher possibility of encephalitic outcomes. The regular instance of human disease due to VEEV infection, the prevalence of mosquito vectors in a global scale and the potential of VEEV to be used as a biological weapon greatly emphasize the need to develop effective post-exposure therapeutic strategies against VEEV [1].

The blood-brain barrier (BBB) is a highly complex, multicellular microenvironment that plays critical roles in controlling bidirectional traffic to and from the central nervous system (CNS) [5,6,7]. The BBB is also a target that is disrupted in the context of multiple proinflammatory states, including virus infection [8,9,10,11]. In VEEV infection, disruption of the BBB and loss of functional integrity are critical parameters that contribute to the encephalitic outcomes [12,13,14]. In cases of aerosol exposure, the virus is known to bypass the BBB to gain access to the CNS, while eventual disruption of the BBB ensues due to inflammatory and viral load in the brain [13]. An effective VEEV therapeutic strategy should address damage to the BBB and mitigation or moderation of the damage so as to prevent long-term disease burden. Traditional two-dimensional models of the BBB, including endothelial trans-well models, do not recapitulate the complex, multicellular architecture of the BBB, thus proving to be poor experimental model systems to address the events that occur at the BBB during viral infection or the effectiveness of therapeutics. Furthermore, current animal models, including the murine, ferret and nonhuman primate models, of VEEV infection do not accurately recapitulate human disease. An even more practical challenge with the utility of the larger animal models is the need for advanced logistical and instrumentational capabilities in higher containment laboratories that do not support robust preclinical screening and product optimization [15]. Therefore, while these model systems greatly support the preclinical product development pipeline for therapeutics development, there is a critical need for the integration of robust, complex in vitro model systems that can be readily deployed in multiple laboratory settings to enable product screening, development and optimization prior to transition to in vivo studies. Inclusion of complex human-relevant models into the pipeline will greatly enhance the translatability of products and increase the potential for clinical success [16,17,18].

In this manuscript, we provide data to support the translational utility of a three-dimensional OOC model system of the human BBB, the neurovascular unit (NVU), in evaluating the antiviral and anti-inflammatory activities of small molecule therapeutic candidates [19,20,21,22]. This model has been previously demonstrated to respond to proinflammatory insults, such as lipopolysaccharide exposure [21]. Specifically, a novel iteration of the NVU that is employed in this study, the gravity-flow NVU (gNVU), is amenable for use in high containment laboratories, including biosafety level 3 (BSL3) level containment that is required to conduct research activities with virulent strains of new world alphaviruses, including VEEV. We demonstrate a proof of concept application of the NVU to assess the antiviral and anti-inflammatory activities of Omaveloxalone (OMA), a small molecule inhibitor that modulates the ubiquitin proteasome signaling pathway. OMA is a highly relevant candidate for potential therapeutic interventions against VEEV, also because its relevance to treat endothelial pathology has been demonstrated; it has been well-studied in animal models, with known pharmacokinetic parameters, and has been evaluated in in-human studies [23,24,25]. We also demonstrate the application of the gNVU platform to assess BBB disruption as influenced by viral virulence and the impact of an effective therapeutic intervention strategy on maintaining barrier integrity. This platform, hence, has significant translational applicability in not only therapeutics development against acutely infectious viruses such as the encephalitic viruses, but also to obtain a mechanistic understanding of cellular events that ensue in the endothelium due to infection and/or inflammation in a manner that takes into account the interactive, multicellular BBB microenvironment.

## 2. Materials and Methods

### 2.1. Gravity NVU (gNVU) Design and Assembly 

**Figure 1:**
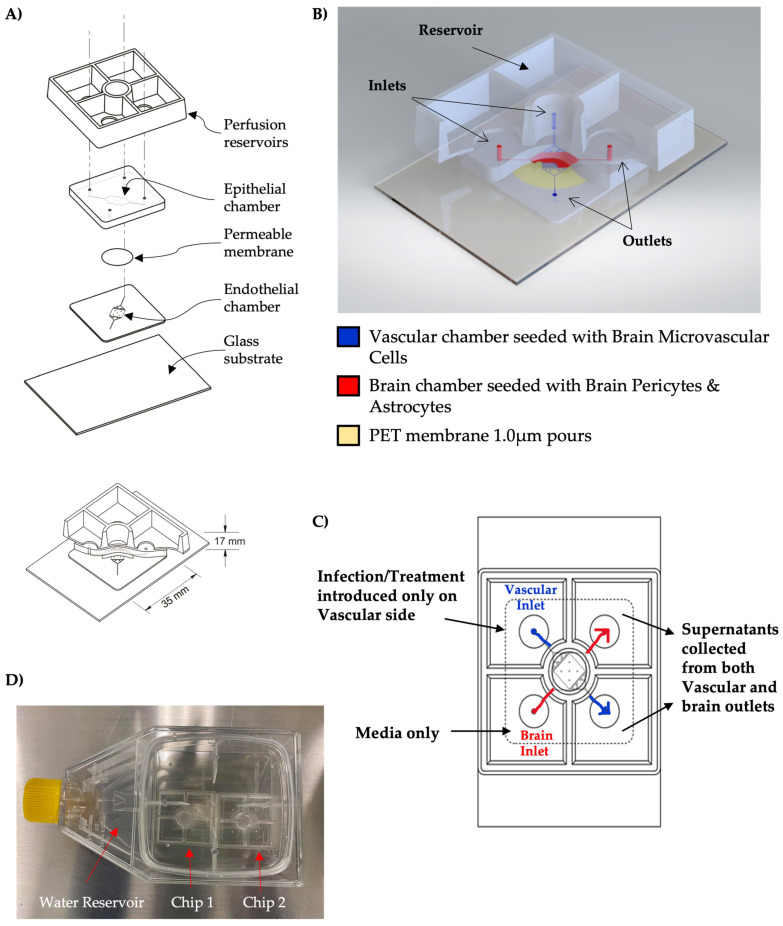
Schematic of the gNVU. (**A**) Artist’s rendering of the gNVU architecture; (**B**) positioning of the inlets and outlets are identified and the loading locations of the endothelial cells, astrocytes and pericytes are indicated; (**C**) the sites of introduction of the infection/therapeutic, control and sample collection sites are indicated. (**D**) Image of gNVU chips in a secondary container safe for use with select agents.

The gNVU schematic and the port layout are illustrated in Figure 1. As previously described in the original NVU model [19,20,21,22], the lamination photolithography technique was used to create positive dry film resist molds whereby PDMS (Poly-dimethylsiloxane, Medical Grade Silastic 7-4814) is cast and subsequently demolded to reveal a negative trough of PDMS features. Feature heights for the dry film resist molds targeted 100 microns each. Two different dry film resists were used, one with higher resolution (Insma PCB DFR), to generate the thin layer with splitters, and one that was lower resolution (Dupont Riston based material GM140 thickness 100 µm), to generate the thick layer without splitters. Four identical layers were spaced out onto each 120 mm × 120 mm mirror-finish polished stainless-steel wafer occupying four equally spaced quadrants. Stainless-steel wafers that have the positive cast features of DFR were placed into a 120 mm × 120 mm square petri dish (Greiner petri dish Cat.688161) to hold the PDMS during oven cure. CAD designed square 120 mm × 120 mm mold spacer holders were laser cut out of 0.1875″ McMaster-Carr acrylic. Onto these acrylic mold spacer holders were four Di-Chloroethane glued spacers, each of two thicknesses, depending on the layer being cast. The thin layer cast used sanded to thickness 0.75 mm spacers from 0.0625″ material (McMaster-Carr 8560K172), one per corner of the outermost square laser cut acrylic spacer holder. The thick layer cast used sanded to thickness 0.1875″ acrylic (Cat. 8774K24) also in each outermost corner of the acrylic spacer holder. A hole was drilled and tapped to accommodate the use of a knurled thumb screw for PDMS–acrylic adhesion reduction when letting down the spacer acrylic layer onto uncured PDMS, and when demolding the devices following a 65 °C four-hour bake. PDMS was poured into each dish to achieve a specified thickness depending on the layer type. Each mold cast required a specific amount of 1:10 ratio mixed PDMS. The thin layer (splitters-containing layer) required 30 g and the thick layer (no splitters layer) required 72 g. Once the PDMS was poured onto the wafer inside the Greiner dish, the thin features required the thin spacer acrylic top mold and the thick features required the thick spacer acrylic top mold. Excess PDMS was squeezed outside above the acrylic top mold spacer holder, such that all that remained between the top acrylic and the bottom stainless-steel feature covered wafer was the PDMS thickness, dictated by the spacers size (0.75 mm or 4 mm thickness). Following PDMS excess push out after placing on the lid, a 1/16″ laser cut acrylic weight positional constraining layer was placed on top of the mold top such that the spacers were held in place and did not shift off the desired spacer contact point per each 200 g weight. Each mold has four 200 g weights placed on top of the acrylic spacer lid at the area where the spacers are glued in place in each corner to ensure coplanarity between the bottom of the acrylic and the top of the stainless-steel wafer prior to the PDMS curing in the oven. The uncured PDMS layers were placed inside a 65 °C curing oven for at least four hours to fully cure the PDMS. Removal of the cured layers required destruction of the petri dish. First the weights were torn out of the cured PDMS and the acrylic positional constraint layer was removed. The knurled screw was tightened in a clockwise direction so that as it pushes downward on the stainless-steel wafer, the acrylic top mold can be removed and unstuck from the cured PDMS top side. The excess PDMS can be torn off, leaving only the desired features intact, and then this 120 mm × 120 mm four feature containing layer can be removed from the stainless-steel wafer. These layers were then cut into fourths, yielding four feature layers each of thick or thin thickness per mold cast. Thick splitterless layers that have been cut out into individual parts were punched at each one of four intubation areas. The thin splitter-containing layers were bonded to a 2″ × 3″ glass microscope slide. A 0.2% solution of Bis Amino {bis(3-trimethoxysilyl)propyl)amine)} in isopropanol with 0.1% deionized (DI) water was placed on an 80 °C hotplate. PET membranes that were cut out of the trans-well housings on an Epilog Laser Cutter were placed into slits cut into a 3″ puck of 4 mm thick PDMS. This PDMS puck membrane holder was then placed into a Harrick benchtop plasma cleaner with the membranes standing up for 40 s of plasma treatment on each side of the membrane. The membranes were then placed into the 0.2% Bis Amino for 10 min. PET membranes were then washed with Isopropanol and placed onto a mylar strip covered glass slide and then placed into a 65 °C oven to dry for thirty minutes. PET membranes were next placed into a 3″ square petri dish filled with 70% ethanol for thirty minutes. The PET membranes were then dried with N2 using a blow gun prior to plasma treating the PDMS layers for final bond completion. The two PDMS layers, thin bonded to glass and thick only punched, were plasma treated in the Harrick plasma cleaner for 40 s features sides up, simultaneously. The PET membrane was bonded first to the thin PDMS side which was already bonded to the microscope slide. The microscope slide was used as a transparent handle for alignment, such that when flipped upside down, the features can be precisely aligned before joining the plasma treated layers together. Following this bond, the bonded device was placed in a 65 °C oven overnight.

### 2.2. NVU Reservoirs

To fabricate passive perfusion reservoirs, a two-part mold set was first machine built in PMMA (McMaser Carr, Atlanta, GA, USA). Each mold set consists of a mold with cavities that form the walls and floor of the reservoirs and a blank plate that caps the mold and forms the bottom face of the reservoirs. The plate was offset 1 mm from the cavity mold to yield an optically clear reservoir floor of the same thickness. Sylgard 184 PDMS (Dow Corning, Midland, MI, USA) polymer was mixed at a 10:1 ratio (base:curing agent) by weight using an AR-100 Planetary Centrifugal Mixer (Thinky, Tokyo, Japan). The mixture was poured into the cavity mold and placed under vacuum for 10 min to pull air bubbles from the liquid. The plate with spacers was then lowered onto the surface of the casting to displace both air and liquid until the spacers were in contact with both parts of the mold. The polymer was allowed to cure at room temperature for at least 48 h, then was demolded and trimmed. A Ø12 mm biopsy punch (Difa Cooper, Milan, Italy) was used to punch a hole in each quadrant of the reservoir’s floor, which allows the contained liquid to access the chip’s fluidic conduits. Finally, the top surface of the NVU chip and the bottom surface of the reservoir were activated with a BD-20AC Laboratory Corona Treater (Electro-Technic Products, Chicago, IL) for 30 s, placed in contact with one another and the assembly was baked at 65 °C for a minimum of 4 h.

### 2.3. Cell Types and Culture

Primary human brain-derived microvascular endothelial cells (HBMVEC, Cat.#ACBRI 376) were procured from Cell Systems (Kirkland, WA, USA) and were maintained in endothelial basal medium (EBM)-2 (CC-3162, Lonza, Basel, Switzerland) containing 5% fetal bovine serum (FBS) and the manufacturer’s growth bullet kit. Human brain vascular Pericytes (HBVPs, Cat.#1200) were obtained from ScienCell (Carlsbad, CA, USA) and astrocytes SVGp12 (CRL-8621) were obtained from American Type Culture Collection (ATCC, Manassas, VA, USA) and were maintained in Eagle’s minimum essential medium, EMEM (ATCC 30-2003), supplemented with 10% heat-inactivated FBS. All cells were maintained in T-25 or T-75 flasks under a standard culture procedure and maintained in 37 °C, 5% CO2 culture conditions.

### 2.4. Cell Loading and Cell Culture in the NVU Device

Prior to cell seeding, NVU chips were vacuumed for 24 h. The following day, chips were treated with ECM (extracellular matrix) of a 5:4:1 mix (distilled ultrasense water, collagen, fibronectin). To load the chips, a 1 mL syringe was first loaded with ECM and then attached with Tygon tubing. Holding the syringe vertically with the needle up, the blunt needle was attached to port 1 of the device. Tilting the device upwards away from the injection port at about 30 degrees, the chamber was filled slowly, avoiding formation of bubbles. The vascular chamber was filled prior to the brain chamber and loaded devices were maintained at 37 °C until ready for use.

#### 2.4.1. Seeding of Endothelial Cells in the NVU

Trypsinized HBMVECs were added to 4 mL EBM-2 medium and spun down. The cells were included in 1 mL EBM-2 medium at a density of 1–2 × 10^6^ cells/mL. Using a 1 mL sterile syringe with a needle blunt, the needle was first filled with medium. The needle was then pushed through the open port valve 1 to slowly inject ~100 µL HBMVECs into an empty device, taking care to avoid introducing bubbles. The device was inverted overnight to let cells attach or for 4–24 h. Vascular media was perfused into the device the next day.

#### 2.4.2. Loading of Astrocytes and Pericytes in the NVU

Trypsinized cells were resuspended in 4 mL EBM-2 medium and spun down. The cells were then resuspended in 1 mL EBM-2 medium at a density of 1–2 × 10^6^ cells/mL. Using a 1 mL sterile syringe, the needle blunt was pushed through the brain port to slowly inject ~100 µL astrocytes and pericytes, taking care to avoid introducing bubbles. After letting cells attach for 2–24 h, the flow was restarted. The following day, flow was checked in both chambers prior to the start of infection experiments.

Each fully assembled gNVU contains ~100,000 endothelial cells, ~100,000 astrocytes and ~50,000–100,000 pericytes.

### 2.5. Viruses and Infection

The live-attenuated VEEV-TC83 strain was obtained from BEI Resources (Venezuelan Equine Encephalitis Virus, TC-83, NR-63). The virulent Trinidad Donkey strain, VEEV TrD was graciously provided by Dr. Kylene Kehn-Hall (current affiliation, Virginia Polytechnic Institute State University). All experiments involving VEEV TrD were carried out in the biosafety level 3 (BSL3) laboratories at the George Mason Biomedical Research Laboratory, in compliance with regulatory and safety requirements. The infection was carried out by introducing the virus through the vascular side port of the NVU by adding virus to the perfusion medium to reach a multiplicity of infection (MOI) of 0.1. The virus-containing medium was continuously perfused into the gNVU for 1 h at 37 °C, 5% CO_2_ conditions. After 1 h of introducing the virus into the gNVU unit, medium with or without inhibitors was perfused into the gNVU and the infected units were maintained at 37 °C, 5% CO_2_ until the termination of the study.

### 2.6. Inhibitors and Drug Treatment

OMA was purchased from MedChemExpress (Omaveloxolone/RTA 408, Cat. HY-12212), solubilized in DMSO and stored in −80 °C as single-use, master stock aliquots. When required, working stocks were made by diluting the master stock in DMSO to attain desired dilutions. To treat the NVUs with the drug, appropriate dilutions of the master stock were added to the cell culture medium, which was perfused into the vascular side of the NVU. The drug was added to the vascular side once every 24 h unless indicated otherwise. The treated NVUs were maintained at 37 °C, 5% CO_2_ following the demands of the experiment until the termination of the study.

### 2.7. Cell Viability Assay

Cell viability assay was performed on inhibitor-treated endothelial cells, astrocytes and pericytes to quantify cell survival using the Cell Titer Glo Cell Luminescent Viability Assay following the manufacturer’s instructions (Promega, G7570, Madison, WI, USA) and as described previously [26,27,28,29,30]. Percent viability of drug-treated cells is represented relative to the DMSO control.

### 2.8. Plaque Assay

Quantification of infectious virus titer by plaque assay was performed following a standardized, published assay protocol [26,27,28,29,30]. Briefly, Vero cells were seeded in 12-well plates at 1.5 × 10^5^ cells per well. Culture supernatants were diluted in DMEM from 10^1^ to 10^8^ and the virus-containing medium was overlaid on the cells for 1 h to permit adsorption and infection. At 1 h post infection, 1 mL of a 1:1 solution of 1% agarose in distilled H_2_O with 2x Eagle’s minimal essential medium was added to the wells and allowed to solidify at room temperature. The plates were subsequently transferred to 37 °C, 5% CO_2_ culture conditions and maintained for up to 48 h. After 48 h, the plates were fixed with 10% formaldehyde overnight at room temperature. Approximately 24 h after fixation, the agar plugs were discarded and fixed cells were stained with 1% crystal violet in 20% methanol solution for 15 min. The plaques were counted for each plate and plaque forming units/mL (PFU/mL) for each sample were calculated. The mean and standard deviation were determined using the average of 3 replicates for each sample.

### 2.9. Blood Brain Barrier Permeability Assay

We used 3kD FITC dextran to evaluate passive permeability (ThermoFisher Scientific). Stock solutions were made in water at 1 mM and stored at −20 °C. Working concentrations were prepared at 1 µM in HBMEC culture media. The vascular and brain compartments were perfused via gravity, achieving an average flow rate of 2 µL/mL over a 24-h period. For passive permeability measurements, effluent was collected from the brain compartment every day 24 h after last renewal of inlet media and analyzed for fluorescence intensity using a plate reader (GloMax Microplate Reader, Promega). By measuring concentration in the brain compartment, the applied permeability coefficient was calculated as:

Papp = Vb × CaCb × A × tPapp = Vb × CaCb × A × t

where V_b_ is the brain chamber volume in cm^3^, A is the membrane growth area in cm^2^, C_a_ is the initial vascular concentration of dextran in µM, C_b_ is the brain concentration of dextran in µM and t is the assay time in seconds. The effective permeability of the BMEC monolayer was calculated by subtracting the permeability of an empty device according to the equation:

1Ptotal = 1Pcells + 1Pmembrane.1Ptotal = 1Pcells + 1Pmembrane

### 2.10. Proinflammatory Cytokine Quantification Assay

Proinflammatory cytokine levels in the perfused media on the brain and the vascular sides of the gNVU were quantified using a commercially available, multiplex cytokine assay kit. Media were collected at the desired time points post infection and stored at −80 °C until ready for the multiplexed ELISA run. The samples were assayed with MSD V-PLEX Proinflammatory Panel Human Kit (Cat. K15049D-2) as duplicates for 10 cytokines: IFN-γ, IL-1β, IL-2, IL-4, IL-6, IL-8, IL-10, IL-12p70, IL-13 and TNF-α. The assay was performed following the manufacturer’s protocol and read using MESO QuickPlex SQ 120 (MesoScale Discovery, Gaithersburg, MD, USA).

### 2.11. Statistics

All quantifications were performed by incorporating data obtained from triplicate samples unless indicated otherwise. Error bars in all figures indicate standard deviations. Plaque assay and ELISA data calculations were performed using Microsoft Excel. Graphs and *p*-values were designed and calculated using unpaired two-tailed t-tests in GraphPad Prism version 9.2.0 for Windows 10 or 9.4.0 for MacOS. Significance values are indicated using One-way ANOVA with Dunnett’s post-test using asterisks as * *p* < 0.05, ** *p* < 0.01, *** *p* < 0.001, **** *p* < 0.0001, or using unpaired two-tailed *t*-test * *p* < 0.05, ** *p* < 0.01, *** *p* < 0.001, **** *p* < 0.0001.

## 3. Results

### 3.1. OMA Decreases Viral Load in the Individual Cellular Components of the NVU in the Context of VEEV-TC83 Infection

**Figure 2:**
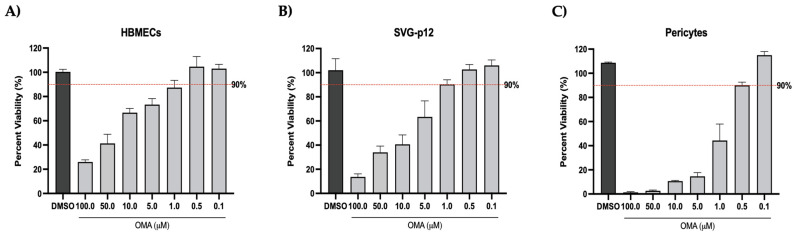
Cell viability assessments of the cellular components of the gNVU following OMA treatment. HBMECs (**A**), SVG-p12 astrocytes (**B**) or pericytes (**C**) were seeded in 96-well plates and allowed to reach confluency overnight. Culture medium with OMA included at increasing concentrations was added to the cells and the cells were cultured at 37 °C for up to 24 h, after which cell survival was measured by CellTiterGlo assay. The % viability of the drug-treated cells is represented relative to the vehicle (DMSO) control. The 90% viability level is indicated by the broken red line. Data were obtained by averaging readings obtained from triplicate samples.

As a first step, infection and drug effectiveness were evaluated in the individual cellular components of the gNVU using the attenuated VEEV-TC83 strain. Prior to assessing the inhibitory potential of OMA, the cytotoxicity of the compound to cells of the gNVU (endothelial cells, astrocytes and pericytes) was determined. The cells were plated individually in a 96-well format and treated with increasing concentrations (0.1–100 µM) of OMA for 24 h. DMSO-treated cells were maintained as reference controls. Cell survival was measured after 24 h of drug treatment by CellTiterGlo assay (Figure 2). In the HBMECs, >90% cell survival was observed at 0.5 µM concentration and a dose–dependent decrease in viability was clearly noted at increasing drug concentrations (Figure 2A). The SVG-p12 astrocytes were slightly more tolerant than the HBMECs and >90% cell survival was noted at the 1 µM concentration (Figure 2B). The pericytes demonstrated >90% survival at the 0.5 µM concentration (Figure 2C). For all experiments involving the fully assembled gNVU, OMA was hence maintained at 0.5µM concentration to ensure that the experiments were performed in the nontoxic range.

**Figure 3:**
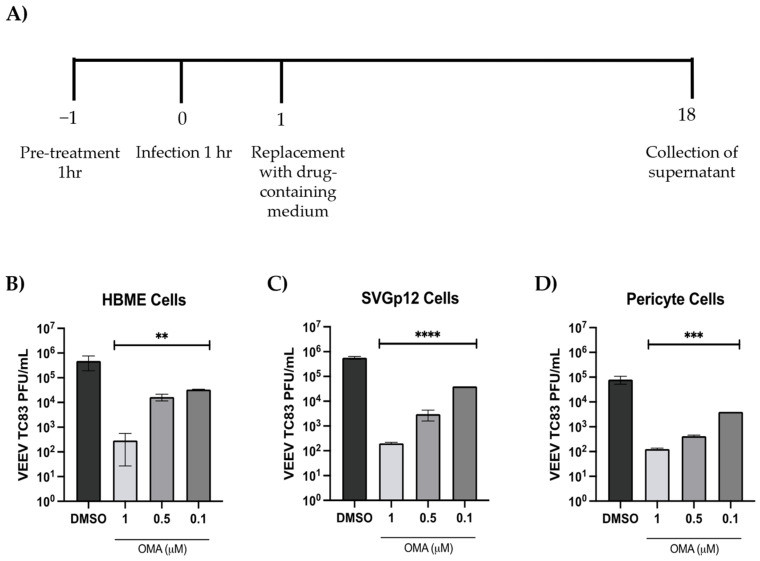
Dose–dependent inhibition of VEEV-TC83 in endothelial cells, astrocytes and pericytes following OMA treatment. (**A**) Schematic representing the dosing and infection strategy. Endothelial cells (**B**), astrocytes (**C**) and pericytes (**D**) were plated in 96-well plates and infected with VEEV-TC83 (MOI:0.1). These cells were pretreated with culture medium containing increasing concentrations of OMA for 1 h prior to infection. After infection, the drug-containing media were added back to the cells and cells were maintained at 37 °C for 18 h. Culture supernatants were obtained at 18 h and viral load was quantified by plaque assay. Infectious viral titer is indicated as plaque forming units (PFU)/mL relative to the DMSO control sample. Data represent the average of three independent samples. Statistical analysis was performed using One-way ANOVA with Dunnett’s post-test. ** *p* < 0.01, *** *p* < 0.001, **** *p* < 0.0001.

As the next step, the responsiveness of the individual cell types to OMA treatment was quantified by pretreating the cells with the inhibitor for 1 h, after which the infection was carried out with VEEV-TC83 (MOI: 0.1) for 1 h. After infection, the infection overlay was removed and replaced with drug-containing medium. The cells were maintained at 37 °C, 5% CO_2_ culture conditions for 18 h, after which the supernatants were collected and virus load was quantified by plaque assay (Figure 3A). In HBMECs, the effective concentration of 0.5 µM resulted in >1 log decrease (Figure 3B). In the astrocyte cells, the 1 µM dose resulted in >3 log reduction of virus titer (Figure 3C). In the pericytes, treatment with 0.5 µM OMA resulted in ~2 log reduction of infectious virus titer (Figure 3D). Cumulatively, the data show that the endothelial cells demonstrated the lowest decrease while the astrocytes showed the highest decrease in infectious viral load following treatment with OMA.

### 3.2. Treatment with OMA Preserves BBB Permeability and Decreased Viral Load in the Brain and Vascular Sides of the gNVU in the Context of VEEV-TC83 Infection

**Figure 4:**
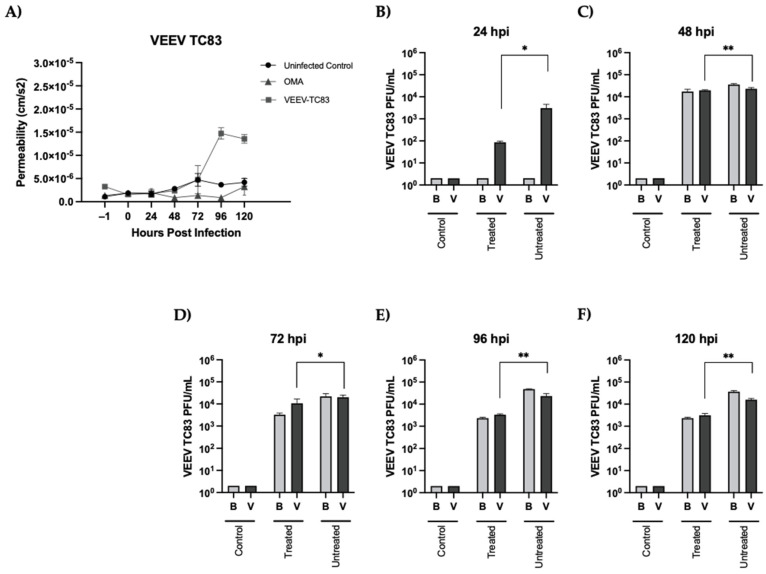
Impact of OMA treatment on BBB permeability and viral load in the brain and vascular compartments of the gNVU in the context of VEEV-TC83 infection. (**A**) Permeability of the endothelial layer of the gNVU was assessed by quantification of FITC-dextran levels in the brain and vascular chambers of the gNVU. Units were either infected with VEEV-TC83 and untreated or infected and treated with OMA. Control units refer to gNVUs that were neither infected nor treated with OMA. Data are obtained as averages from *n* = 3 each for infected, treated and infected, untreated sample, and *n* = 2 for control samples; (**B**–**F**) Longitudinal quantification of viral load in the brain (B) and vascular (V) sides of the NVU following infection by VEEV-TC83 (infected) and treatment with OMA (treated). Control refers to NVUs that were neither infected nor treated. Data are obtained as averages from *n* = 3 each for infected, treated and infected, untreated samples, and *n* = 2 for control samples, and infectious titers are represented as PFU/mL. Statistical analysis was performed using One-way ANOVA with Dunnett’s post-test. * *p* < 0.05, ** *p* < 0.01.

The effectiveness of OMA to protect the integrity of the BBB and decrease the viral load in the brain and vascular compartments was quantified using infected gNVUs. Briefly, OMA-containing culture medium was perfused into the gNVU for 1 h prior to the infection and the NVU was maintained in the presence of drug at 37 °C, 5% CO_2_ conditions. Medium containing VEEV-TC83 (MOI: 0.1 as calculated based on endothelial cell number per mL) was introduced into the vascular inlet of the gNVU and the unit was transferred to the incubator for 1 h. After 1 h to permit infection, OMA-containing medium was reintroduced into the gNVU and the unit was maintained at 37 °C, 5% CO_2_ conditions for up to 120 h. Drug-containing medium was added once every 24 h to each gNVU unit. gNVUs which did not receive any drug treatment or infection were maintained as no-infection-controls throughout the experiment. The endothelial integrity as inferred based on permeability of FITC-dextran was quantified once every 24 h for the total 120 h (Figure 4A). In the infected, untreated NVUs, a notable increase in permeability was observed after the 72-h time point, with a statistically significant, prominent increase in permeability noted at the 96-h time point. In stark contrast, the infected NVUs that were treated with OMA (0.5 µM) maintained the integrity of the barrier in spite of the infection, to levels that were comparable to the uninfected, untreated control gNVUs. This dataset demonstrates that OMA treatment confers protection to the integrity of the BBB and minimizes, if not mitigates, the infection-induced disruption.

Perfused media were obtained from both the brain and the vascular sides once every 24 h and longitudinal virus load in each gNVU was quantified by plaque assay (Figure 4B–F). At the 24-h time point, the viral load on the brain side of the infected, untreated or the infected, treated gNVU was indistinguishable from the uninfected control (Figure 4B). The viral load on the vascular side of the infected, untreated gNVU showed >10^3^ PFU/mL of infectious virus. In the infected, treated gNVU, a statistically significant decrease in viral titer was noted (>1.5 log) on the vascular side. Between the 24- and 48-h time points, the viral load in both the brain and vascular side of the infected, untreated and infected, treated gNVUs reached ~10^4^ PFU/mL, with only a modest, but statistically significant decrease in viral titer noted in the vascular side (Figure 4C). Between the 72 and 120-h time points, no further increase in viral load was noted in the brain and vascular sides of the infected, untreated gNVUs, suggesting that the infection had stabilized within that window of study (Figure 4D–F). The viral load began to drop in the brain and vascular sides of the infected, treated gNVU at the 72-h time point, which further decreased at the 96-h time point and stabilized, with no further change (increase or decrease) noted at the 120-h time point. Overall, the longitudinal analysis of the infection dynamics on the brain and vascular sides of the gNVU in the presence of OMA indicated that drug treatment effectively decreases viral load very early in the infection of the gNVU and subsequently maintains a lower viral load for up to 120 h post infection when compared to the infected, untreated gNVUs.

### 3.3. OMA Treatment Decreases the Proinflammatory Cytokine Load in the Brain and Vascular Compartments of the VEEV-TC83-Infected gNVUs

**Figure 5:**
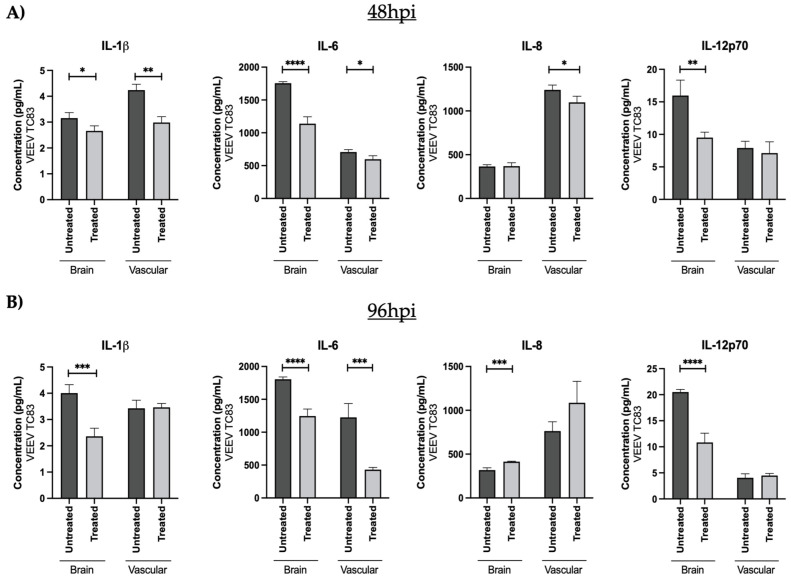
Quantification of proinflammatory cytokine load in the brain and vascular compartments of the gNVU following VEEV-C83 infection and treatment with OMA. The perfused media from the brain and the vascular sides of the gNVUs (infected, untreated [infected][*n* = 3], infected, treated [treated][*n* = 3] were collected at 48 h post infection (**A**) and 96 h post infection (**B**). The amounts of 10 proinflammatory cytokines were analyzed in these samples by multiplexed ELISA, of which data for 4 cytokines (IFN-γ, TNF-α, IL-1β, IL-6, IL-8 and IL12-p70) are included. Each sample was included in the multiplexed ELISA as technical duplicates and the data were averaged for each analyte at each time point. The overall average of the biological replicates was obtained and represented as pg/mL concentration for each cytokine. Statistical analysis was carried out using *t*-test * *p* < 0.05, ** *p* < 0.01, *** *p* < 0.001, **** *p* < 0.0001.

OMA is well-documented to be an activator of Nrf2, which in turn elicits suppression of inflammatory outcomes. The impact of OMA treatment on the proinflammatory cytokine levels of the VEEV-TC83-infected gNVUs was then assessed by multiplexed ELISA for ten candidate cytokines using the same perfusion media as used for viral quantification in (Figure 4). These measurements were carried out at the 48- and 96-h timepoints post infection. The data included in Figure 5 show the outcomes on the brain and vascular compartments for six proinflammatory cytokines and additional data are included in Appendix A. Higher levels of IFN-γ, IL-6 and IL-12p70 could be noted on the brain side of the gNVUs at both time points, suggesting that these may be proinflammatory markers in the brain side (Figure 5A,B). In contrast, higher levels of IL-1β and IL-8 were noted in the vascular side of the gNVU at both time points, suggesting these to be candidate proinflammatory markers in the vascular side. TNF-α levels, however, demonstrated only a modest change at both time points.

In the context of OMA treatment, a statistically significant decrease in the vascular side markers (TNF-α, IL-1β and IL-8) could be observed at the 48-h time point (Figure 5A). While a decrease in TNF-α and IL-8 could be observed in the vascular side of the OMA-treated gNVUs at the 96-h time point, there was not an observable statistical significance to this outcome. IL-1β levels showed no change between the treated versus untreated gNVUs in the vascular side at the 96-h time point. OMA treatment, therefore, resulted in the decrease of three important proinflammatory cytokine markers in the vascular side of the infected gNVU.

When looking at the brain side proinflammatory markers, IL-6 and IL-12p70 showed a statistically significant, notable drop at the 48- and the 96-h time points. The IFN-γ levels also demonstrated a strong drop at the 96-h time point. Hence, at the later time point of 96-h post infection, OMA was able to decrease the levels of three prominent proinflammatory cytokine markers in the brain side of the infected gNVU.

### 3.4. OMA Treatment Decreases Infectious Virus Titer and Proinflammatory Cytokine Load in gNVUs Infected with VEEV-TrD

**Figure 6:**
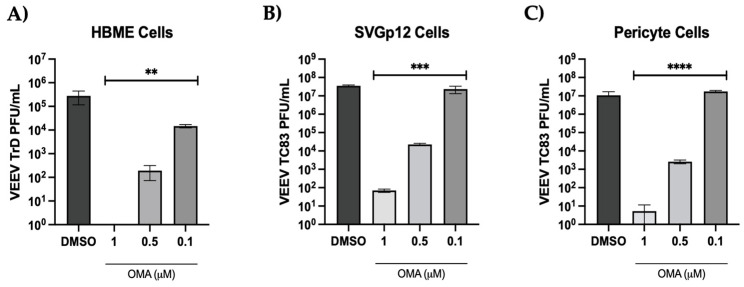
Dose–dependent inhibition of VEEV TrD in the endothelial cells, astrocytes and pericytes. Endothelial cells (**A**), astrocytes (**B**) and pericytes (**C**) were seeded in 96-well plates. The cells were pretreated with increasing concentrations of OMA for 1 h prior to infection. DMSO was maintained as the vehicle-alone control. The drug-containing media were removed and virus-containing media (TrD at MOI: 0.1) were added to the cells for 1 h to permit infection. The virus-containing medium was replaced with drug-containing media after 1 h and cells were maintained at 37 °C for 18 h. Culture supernatants were analyzed by plaque assay and infectious titer is represented as PFU/mL. Data represent the average of three independent samples. Statistical analysis was performed using One-way ANOVA with Dunnett’s post-test. ** *p* < 0.01, *** *p* < 0.001, **** *p* < 0.0001.

The impact of OMA treatment in the endothelial cells, astrocytes and pericytes in the context of VEEV-TrD infection was first evaluated for each cell type individually. Cells were pretreated with inhibitor for 1 h, infected with VEEV TrD (MOI: 0.1) for 1 h and infection overlay replaced with drug-containing media. The supernatants from the drug-treated and the DMSO-treated cells were obtained at 18 h post infection and analyzed by plaque assay. In all three cell types, ≥ 3 log decrease in TrD titers could be noted at the 0.5 uM concentration of OMA (Figure 6), demonstrating that OMA treatment could achieve a consistent decrease in viral load even in the context of infection by the virulent VEEV strain.

**Figure 7:**
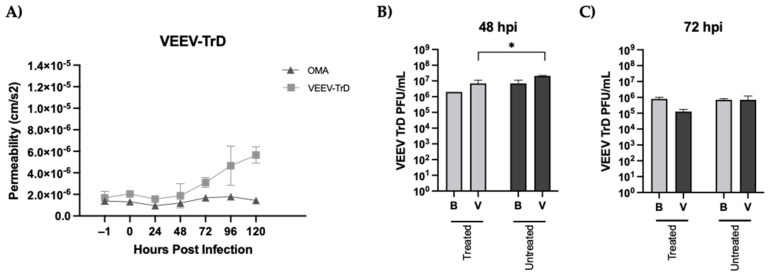
Impact of OMA treatment on the BBB permeability and viral load in the gNVU in the context of VEEV TrD infection. (**A**) gNVUs that were infected (TrD[MOI:0.1]), untreated (*n* = 3) or infected and treated with OMA (0.5 µM) (*n* = 3) were assessed for endothelial permeability status by quantification of FITC-dextran in the brain and vascular chambers. Permeability is depicted as variables of porous membrane units and time (cms/s^2^) and is the average of three independent data points for each time point represented; (**B**,**C**) the perfused media obtained from the brain and vascular compartments of the infected, untreated and infected treated gNVUs at 48- and 72-h post infection were used to quantify infectious viral titer by plaque assay. Data represent average of three independent samples and titers are expressed as PFU/mL. Statistical analysis was carried out using *t*-test * *p* < 0.05.

The impact of OMA treatment on the BBB permeability and viral load in the brain and vascular compartments was next quantified using the assembled gNVUs as described earlier for VEEV-TC83. In contrast to VEEV-TC83, a statistically significant and noticeable change in permeability could be observed as early as the 72-h time point in the context of the TrD infection, which continued to steadily increase up to the 120-h mark (Figure 7A). OMA treatment, however, was able to mitigate this permeability change in the TrD-infected NVUs, with no noticeable change observed in the endothelial permeability to FITC dextran over the entire 120-h time frame. The viral load quantification on the brain and vascular sides at the 48-h time point showed only a very modest, yet statistically significant decrease in the viral titer in the context of OMA treatment as compared to the infected, untreated NVU. Importantly, in contrast to the inhibition of viral titer observed with OMA treatment at the 72-h time point with the VEEV-TC83 infection, gNVUs infected with TrD and treated with OMA did not demonstrate any statistically significant change in viral load in either compartment when compared to the infected, untreated control (Figure 7B). 

**Figure 8:**
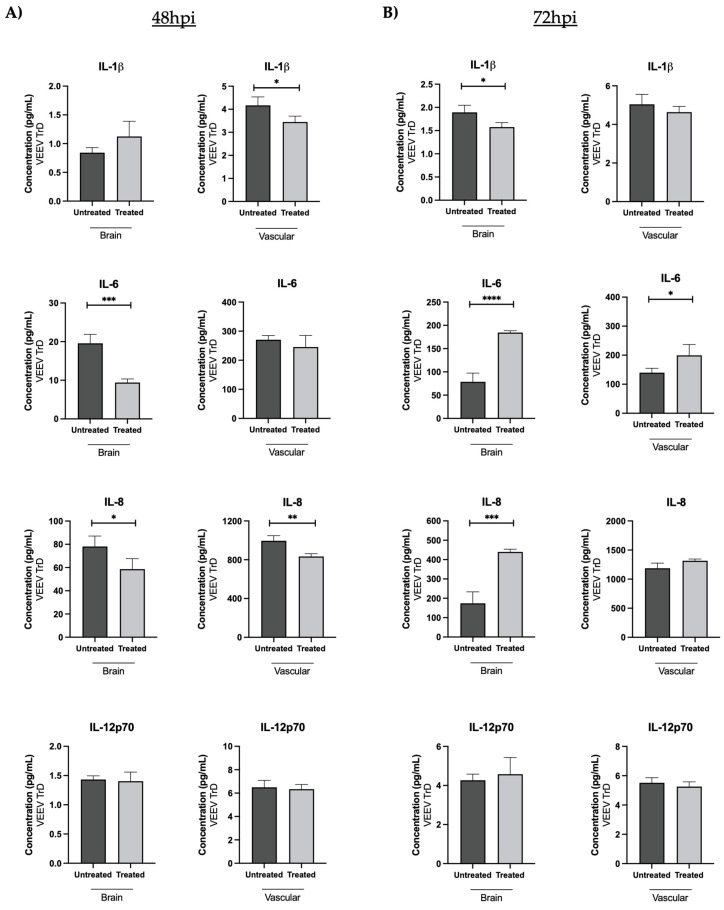
Effect of OMA treatment on proinflammatory cytokines in the gNVU in the context of VEEV TrD infection. Perfused media from the brain and vascular compartments of the gNVUs that were either infected, untreated (untreated) or infected, treated (treated) obtained at 48 h (**A**) or 72 h (**B**) post infection were assessed by multiplexed ELISA for the levels of 10 proinflammatory cytokines. Four of the cytokines that demonstrated outcomes with statistical significance at either of the time points are included here (IFN-γ, IL-1β, IL-6, IL-8). Each sample was queried as technical duplicates in the ELISA and the data were averaged for each analyte at the two time points. The overall average of the biological replicates was obtained and represented as pg/mL concentration for each cytokine. Statistical analysis was carried out using *t*-test * *p* < 0.05, ** *p* < 0.01, *** *p* < 0.001, **** *p* < 0.0001.

The proinflammatory cytokine load on the brain and vascular sides of the gNVU following OMA treatment was quantified as compared to the infected, untreated gNVUs. Treatment resulted in a statistically significant decrease in IL-1β levels in the vascular side and IL-6 levels in the brain side at the 48-h time point (Figure 8A). At the 72-h time point, the IFN-γ, levels decreased in the treated gNVUs on both sides (Figure 8B). The IL-6 and IL-8 levels actually increased in the treated cells at the 72-h time point, which agreed with the data noted in Figure 7C that the viral load increased in the brain side at the 72-h time point regardless of treatment. Overall, the analysis of dynamics of viral and inflammatory load in the context of infection by the virulent strain demonstrated lesser viral inhibition and lower proinflammatory cytokine decrease by OMA as compared to the respective datasets obtained with the attenuated strain.

## 4. Discussion

There is a significant need to develop a robust pipeline of therapeutic candidates that can be eventually successfully translated into treatment strategies against alphaviruses in general, and encephalitic alphaviruses in particular. New world alphaviruses, including VEEV and Eastern Equine Encephalitis Virus (EEEV), are transmitted by arthropod vectors and cause outbreaks in the Americas annually [1,31,32,33,34,35]. Given the disease burden (VEEV, EEEV) and the mortality rate (EEEV, with >50% mortality rate) associated with these viruses, the need to develop effective intervention strategies is paramount. VEEV-TC83 is a live-attenuated strain of VEEV which is used to vaccinate at-risk personnel. However, due to potential safety concerns, in spite of the robust seroconversion noted, VEEV-TC83 is not deployed as a vaccine in civilian populations. Both VEEV and EEEV are highly infectious as aerosols, with broad tissue tropism and higher mortality rates in experimental animal models. When the infection is acquired through the aerosol route, the virus is known to bypass the BBB and establish an infection in the CNS [13], presumably earlier than in the context of mosquito-transmitted infection. The inflammatory burden in the CNS, either early or late in the course of infection and disease in the host, plays an important role in the encephalitic outcomes, resulting in long-term neurological sequelae in survivors. 

A critical challenge and a capability gap in the preclinical therapeutic development pipeline for encephalitic alphaviruses is the availability of relevant model systems that can permit: (a) robust in vitro assessment of lead candidates in a manner that is inclusive of the appropriate tissue microenvironment, (b) human-based platforms that will enable scientific inquiry that will align better with clinical readouts and application strategy, (c) in vivo models that faithfully recapitulate human disease so as to be well-suited for the two-animal-rule regulatory requirement. Conventional approaches to lead discovery and subsequent development rely heavily on two dimensional cultures involving cell lines that are either not human-derived or have been immortalized. Furthermore, these in vitro approaches, while well-suited for high throughput discovery efforts, do not reflect the pathological mechanisms that underlie human disease. Animal models, including murine models, are excellent platforms for early feasibility assessments, but run the risk of prioritizing candidates for large animal studies based on readouts that do not apply to human disease.

OOCs and organoid models have ushered in an era of complex, human-like, human-based, higher order experimental in vitro platforms that, at a minimum, replicate the multicellular architecture of tissues in a manner that is superior to two-dimensional monocultures. These complex organ-on-a-chips are currently being extensively employed to understand human disease, evaluate drug toxicity and assess responsiveness to disruptive stimuli that energize the development of rational and effective intervention strategies [36,37,38,39,40,41,42]. It is therefore a valuable opportunity to develop such complex, tissue-relevant and human-centric experimental platforms as infection models that can be used to understand disease in a multicellular context at the molecular level and to insert a clinically relevant vetting strategy prior to transition to in vivo models. 

The data included in this effort attempt to evaluate the utility of such an OOC platform that reflects the human BBB, referred to as the NVU. The NVU recapitulates the BBB microenvironment by integrating astrocyte- and pericyte-derived cues with the vascular endothelium, thus providing tissue-level complexity [19,20,21,22]. The NVU has been previously employed to describe metabolic signatures related to inflammation following LPS challenge [21]. In this effort, a next-generation NVU, referred to as the gNVU, is described. The gNVU maintains perfusion based on gravity flow, which permits the entire microphysiological unit to be enclosed in a sealed space, thus it is greatly conducive for experiments involving select agent pathogens such as VEEV. Studies involving select agent pathogens such as VEEV and EEEV should be conducted in BSL3 conditions, with pathogen containment being a critical regulatory aspect. The gNVU permits experimentation while maintaining containment, thus being compliant with federal select agent regulations. The data included in this manuscript show that the gNVU can sustain VEEV infection, which can be measured in a longitudinal manner (Figure 4 and Figure 7). The three cell types included in the gNVU can independently be infected by VEEV-TC83 and TrD and respond to small molecule intervention (Figure 3 and Figure 6). As a highly relevant readout, the gNVU responds to the infection through changes in barrier permeability. More significantly, the differential susceptibilities of barrier disruption as related to pathogen virulence can be captured through the gNVU (Figure 3A vs. Figure 7A).

The gNVU also demonstrates discerning capabilities that can differentiate between biomarkers that reflect CNS inflammatory events and biomarkers that can enable efficacy of therapeutic strategies. For example, the brain side of the gNVU showed IFN-γ, IL-6 and IL12-p70 as upregulated cytokines, while the vascular side showed IL-1β and IL-8 as upregulated cytokines in the context of infection by VEEV-TC83 (Figure 5). This dataset also reflects on the anti-inflammatory potential of OMA in the context of this strain as the key cytokines, IL-1β, IL-6 and IL-8 all show decreases. These cytokines, along with others, are strongly associated with organ/tissue damage resulting from inflammatory insult in many infectious disease states [43,44,45,46,47,48]. The ability to differentiate between the origin of these inflammatory signals, i.e., brain versus vascular, will offer insights into the design of inflammatory biomarker diagnostics that can monitor disease progression and therapeutic efficacy. It is important to note that the gNVU is also able to reflect on virulence-related pathologies and the effectiveness of a candidate therapeutic. Case in point: data shown in Figure 8 indicate how OMA, which could effectively control multiple cytokines on the brain and vascular side of the gNVU in the context of VEEV-TC83 infection, was less robust in the context of VEEV-TrD when applied at the same concentration. The extent of virus control in the two compartments was also lower than what was noted for VEEV-TC83. Thus, the gNVU is able to offer a greater level of specificity in the measured outcomes that reflect pathogen virulence, tissue susceptibility and parameters that correspond to mitigation/moderation of disease features.

In its current form, the gNVU does not include neurons. Neurons are important targets of VEEV infection, in addition to the astrocytes and the pericytes. Efforts in gNVU development and evolution are focused on the integration of neurons and microglial cells so that the disease pathology can be studied in the completeness of the CNS immune system. The microglial cells will also be an important addition to assess the role of anti-inflammatory strategies to minimize tissue damage in the context of such explosively inflammatory viral infections.

## 5. Conclusions

Organ-on-a-chips, such as the gNVU, also open attractive avenues to address tissue-level pathobiology, identification of host-based drug targets and evaluate therapeutic candidates against other viral pathogens that are known to have short-term and long-term CNS outcomes, such as polio virus, dengue virus, rift valley fever virus and hemorrhagic fever viruses [45,46,47,48,49,50,51,52,53,54,55,56]. Models such as the gNVU will support translational product development in a clinically relevant context for these pathogens for which the two-animal rule will play an imperative role and animal models are challenging when it comes to recapitulation of human disease.

## Data Availability

Data is contained within the article.

## Appendix A

**Table A1:** Pro-inflammatory cytokines.

Time Point	NVU Chamber	IL-2	IL-4	IL-10	IL-13	IFN-γ	TNF-α
48hpi VEEV TC83	Brain	ns	ns	ns	ns	ns	ns
	Vascular	ns	ns	ns	ns	ns	0.0087 **
96hpi VEEV TC83	Brain	ns	ns	ns	ns	<0.0001 ****	ns
	Vascular	ns	ns	ns	ns	<0.0001 ****	0.0101 *
48hpi VEEV TrD	Brain	ns	ns	ns	ns	ns	ns
	Vascular	0.0055 **	ns	ns	ns	ns	ns
72hpi VEEV TrD	Brain	ns	ns	ns	ns	0.0287 *	ns
	Vascular	0.0098 **	ns	ns	ns	0.0253 *	ns

Statistical analysis was carried out using *t*-test * *p* < 0.05, ** *p* < 0.01, **** *p* < 0.0001, ns = not significant.
